# Efficacy of fasudil in COPD-associated pulmonary arterial hypertension: meta-analysis of randomized controlled trials

**DOI:** 10.3389/fmed.2026.1723597

**Published:** 2026-01-26

**Authors:** Pei Shu, Guorui Xu, Yuling Liu, Ni-Ni Qu

**Affiliations:** 1 Liaoning University of Traditional Chinese Medicine, Shenyang, Liaoning, China; 2 The First Affiliated Hospital of Liaoning University of Traditional Chinese Medicine, Shenyang, Liaoning, China; 3 First Clinical College of Liaoning University of Traditional Chinese Medicine, Shenyang, Liaoning, China; 4 Department of Integrated Traditional and Western Medicine, Liaoning Cancer Hospital and Institute, Shenyang, Liaoning, China

**Keywords:** chronic obstructive pulmonary disease, fasudil, meta-analysis, pulmonary arterial hypertension, rho-kinase inhibitor, systematic review

## Abstract

**Background:**

Pulmonary arterial hypertension (PAH) is a serious complication of chronic obstructive pulmonary disease (COPD) that markedly worsens functional capacity and prognosis. Fasudil, a selective Rho-kinase inhibitor, has shown vasodilatory and vascular-protective effects; however, its therapeutic value in COPD-associated PAH has not been systematically quantified.

**Objective:**

The objective of the study was to evaluate the efficacy of fasudil as an adjunctive therapy for COPD patients with PAH through a systematic review and meta-analysis of randomized controlled trials (RCTs).

**Methods:**

Eight electronic databases were searched from inception to April 2024 for RCTs comparing fasudil plus conventional therapy with conventional therapy alone. Primary outcomes included overall treatment effectiveness and pulmonary artery systolic pressure (PASP). Secondary outcomes were blood oxygen saturation (SaO₂), arterial oxygen tension (PaO₂), and 6-min walk distance (6MWT). Data were pooled using fixed- or random-effects models according to heterogeneity.

**Results:**

A total of 11 RCTs involving 865 participants met the inclusion criteria. Fasudil significantly increased the overall effective rate (risk ratio = 1.18, 95% CI = 1.05–1.31, *p* = 0.004) and reduced PASP (mean difference = −9.42 mmHg, 95% CI = −10.73 to −8.12, *p* < 0.001) with negligible heterogeneity. Chronic treatment (≥2 weeks) improved SaO₂ (MD = 3.56, 95% CI 1.73–5.40), whereas single-dose administration had a minimal effect. PaO₂ increased modestly (MD = 2.19 mmHg, 95% CI = 0.84–3.54, *p* = 0.002). Functional capacity improved substantially, with a 51.96-m gain in 6MWT distance (95% CI = 36.84–67.08, *p* < 0.001), exceeding the minimal clinically important difference.

**Conclusion:**

Fasudil confers consistent short-term benefits in COPD-related PAH, significantly lowering pulmonary pressures and enhancing oxygenation and exercise tolerance. While the included studies were of moderate methodological quality and limited to Chinese settings, the pooled evidence supports fasudil as a promising adjunct for managing COPD-associated PAH. Larger, multicenter RCTs with longer follow-up are warranted to confirm its long-term efficacy and safety. The short follow-up (maximum 4 weeks) limits insights into sustained benefits or progression; long-term trials are essential.

## 1 Introduction

Chronic obstructive pulmonary disease (COPD) represents one of the most prevalent and burdensome respiratory conditions worldwide, characterized by progressive airflow limitation and systemic manifestations that extend beyond the lungs. COPD affects >400 million people globally and is the third leading cause of death, with projections rising to 600 million by 2050 (1). In China, epidemiological data revealed an alarming prevalence of 8.2% among individuals over 40 years of age, affecting approximately 100 million people and imposing substantial socioeconomic challenges on the healthcare system (2).

The pathophysiology of COPD involves chronic inflammation of the airways, parenchymal destruction, and structural remodeling, ultimately leading to irreversible airflow limitation (3). This persistent inflammatory cascade, coupled with oxidative stress and protease–antiprotease imbalance, triggers a series of pathological changes, including mucus hypersecretion, ciliary dysfunction, and alveolar destruction (4). Environmental factors play a crucial role in disease development and progression, with emerging evidence demonstrating that air pollution and occupational exposures to dust and chemical irritants significantly increase COPD risk and exacerbate existing disease (5, 6). These environmental insults perpetuate the inflammatory cycle, accelerating disease progression and increasing the likelihood of complications.

Among the various complications associated with COPD, pulmonary arterial hypertension (PAH) represents a particularly serious sequela that significantly impacts prognosis and quality of life. The development of PAH in COPD patients follows a predictable pathophysiological sequence: chronic alveolar hypoxia induces pulmonary vasoconstriction, endothelial dysfunction promotes vascular remodeling, and inflammatory mediators perpetuate vascular injury (7). PAH complicates 30–70% of COPD cases, with a prevalence of ~39% reported in meta-analyses [42]. Studies have demonstrated that moderate COPD frequently presents with subclinical PAH, while severe disease invariably involves some degree of pulmonary vascular involvement (7). This progression from COPD to PAH represents a critical transition point in disease trajectory, as the development of pulmonary hypertension independently predicts mortality and marks the evolution toward cor pulmonale and right heart failure.

Current therapeutic approaches for COPD-associated PAH remain limited and often inadequate. Non-pharmacological interventions, including long-term oxygen therapy and smoking cessation, provide modest benefits but cannot reverse established vascular remodeling (8). Conventional pharmacological options—including angiotensin-converting enzyme inhibitors, endothelin receptor antagonists, and phosphodiesterase-5 inhibitors—are constrained by systemic hemodynamic effects, limited efficacy in the pulmonary circulation, and significant adverse effect profiles (9). This therapeutic gap has prompted investigation into novel targeted therapies that can selectively address pulmonary vascular dysfunction without compromising systemic hemodynamics.

The Rho/Rho-kinase pathway has emerged as a promising therapeutic target in pulmonary vascular disease, with accumulating evidence supporting its central role in vasoconstriction, inflammation, and vascular remodeling (10, 11). Fasudil, a selective Rho-kinase inhibitor and isoquinoline sulfonamide derivative, has exhibited remarkable efficacy in preclinical models and preliminary clinical studies (12). Its mechanism of action involves competitive inhibition of the Adenosine Triphosphate (ATP)-binding site of Rho-kinase, thereby preventing downstream phosphorylation of myosin light chain and reducing vascular smooth muscle contraction (11, 13). Beyond its direct vasodilatory effects, fasudil exhibits pleiotropic actions, including anti-inflammatory properties, endothelial protection, and inhibition of vascular remodeling—all critical processes in PAH pathogenesis (14).

Despite encouraging preliminary data and increasing clinical use of fasudil in COPD-associated PAH, the evidence base remains fragmented, with individual studies limited by small sample sizes and methodological heterogeneity. The absence of systematic synthesis of available evidence has hindered the development of evidence-based treatment recommendations and optimal therapeutic protocols. A recent meta-analysis on fasudil in Group-3 PH, including COPD-PAH, reported similar benefits on PASP and 6MWT [37]. Building on previous research, our COPD-specific focus extends these findings. Therefore, this meta-analysis was undertaken to systematically evaluate the clinical efficacy and safety of fasudil in patients with COPD complicated by PAH, providing a comprehensive assessment of its effects on hemodynamic parameters, oxygenation status, and functional capacity. By synthesizing the available randomized controlled trial evidence, this study aimed to inform clinical decision-making and establish the role of fasudil in the therapeutic armamentarium for this challenging patient population.

## 2 Materials and methods

### 2.1 Study design and protocol registration

This systematic review and meta-analysis was conducted in accordance with the Preferred Reporting Items for Systematic Reviews and Meta-Analyses (PRISMA) guidelines. The protocol was developed *a priori* to minimize bias and ensure methodological rigor. The protocol was not prospectively registered [e.g., International Prospective Register of Systematic Reviews (PROSPERO)], as this was an unfunded academic project.

### 2.2 Literature search strategy

A comprehensive and systematic literature search was conducted across multiple electronic databases, including PubMed, Web of Science, Cochrane Library, Google Scholar, China National Knowledge Infrastructure (CNKI), Wanfang, Embase, and VIP databases, from their inception to April 2024. The search strategy used both Medical Subject Headings (MeSH) terms and free-text keywords, utilizing Boolean operators to maximize sensitivity while maintaining specificity. The primary search terms included: (“fasudil” OR “Rho-kinase Inhibitor”) AND (“chronic obstructive pulmonary disease” OR “COPD”) AND (“PAH” OR “pulmonary arterial hypertension” OR “pulmonary hypertension”).

Search strategies were adapted to comply with the syntax requirements of each database. No language restrictions were imposed initially, although the final inclusion was limited to English and Chinese publications. The reference lists of included studies and relevant systematic reviews were manually searched to identify additional eligible studies. Gray literature was searched through clinical trial registries and conference proceedings.

### 2.3 Study selection criteria

#### 2.3.1 Inclusion criteria

Studies were eligible for inclusion if they met the following criteria:

1. Randomized controlled trials (RCTs), regardless of blinding status (single-blind, double-blind, or open-label).
2. Parallel-group design with both intervention and control arms.
3. Study population comprising patients with a confirmed diagnosis of COPD complicated with PAH or patients with either condition alone.
4. Baseline characteristics (age, gender, GOLD stage, and baseline PASP) exhibiting no statistically significant differences between groups in age, gender, or disease severity, ensuring adequate comparability.
5. Intervention consisting of conventional treatment plus fasudil in the observation group and conventional treatment alone in the control group.
6. Reporting of at least one of the following outcome measures: total treatment effectiveness, blood oxygen saturation (SaO₂), arterial oxygen tension (PaO₂), pulmonary artery systolic pressure (PASP), or 6-min walk test (6MWT) distance.

#### 2.3.2 Exclusion criteria

Studies were excluded based on the following criteria: non-randomized study designs; duplicate publications or overlapping patient cohorts; absence of a control group; animal or *in vitro* studies; unclear or contradictory methodology, results, or conclusions; obvious errors in statistical methods or data analysis; incomplete experimental design or inability to extract essential data; publications by the same author on similar topics (only the most comprehensive study was retained); absence of common outcome indicators with other included studies; inclusion of patients with serious comorbidities affecting the heart, liver, kidney, brain, or hematological system; and publications in languages other than Chinese or English.

### 2.4 Study selection and data extraction process

Two investigators independently performed study selection following a standardized two-stage screening process. Initial screening involved reviewing titles and abstracts to identify potentially eligible studies, followed by full-text assessment of selected articles against the predetermined inclusion and exclusion criteria. The screening process was facilitated using NoteExpress reference management software to organize retrieved citations and identify duplicates. Disagreements between reviewers were resolved through discussion, with a third investigator consulted when consensus could not be reached.

Data extraction was performed independently by two investigators using a standardized, pre-piloted data extraction form. The form was tested on three randomly selected studies and refined before full implementation. Extracted data encompassed study characteristics (title, authors, and publication year), methodological details (randomization method, allocation concealment, and blinding), participant demographics (sample size per group and baseline characteristics), intervention parameters (fasudil dosage, route of administration, and treatment duration), and outcome measures with their corresponding statistical data. When data were presented only in graphical format, digital extraction tools were used to obtain numerical values. Authors of primary studies were contacted via email when clarification or additional data were required, with a maximum of three contact attempts over 4 weeks. Cohen’s kappa for agreement was 0.85.

### 2.5 Risk of bias assessment

Methodological quality and risk of bias were evaluated using the Cochrane Collaboration’s risk of bias tool, which assesses six key domains: random sequence generation (selection bias), allocation concealment (selection bias), blinding of participants and personnel (performance bias), blinding of outcome assessment (detection bias), incomplete outcome data (attrition bias), and selective reporting (reporting bias). Each domain was judged as having low risk, unclear risk, or high risk of bias according to predetermined criteria outlined in the Cochrane Handbook for Systematic Reviews of Interventions.

Two reviewers independently assessed each study, with disagreements resolved through discussion or arbitration by a third reviewer. Overall study quality was classified into three categories: Grade A (low risk of bias across all domains), Grade B (unclear risk in one or more domains), and Grade C (high risk of bias in one or more domains). Inter-rater reliability was assessed using Cohen’s kappa coefficient. Kappa was 0.82.

### 2.6 Statistical analysis

All statistical analyses were performed using Review Manager (RevMan) version 5.4 software. For continuous outcomes, weighted mean differences (MD) with 95% confidence intervals (CI) were calculated, while dichotomous outcomes were analyzed using relative risk (RR) with 95% CI. The choice between fixed-effects and random-effects models was determined *a priori* based on statistical heterogeneity assessment. Certainty of evidence was assessed using the GRADE approach for each outcome, starting as ‘High’ for RCTs and downgraded for risk of bias, inconsistency, indirectness, imprecision, and publication bias. The results are presented in Supplementary Table 1.

Statistical heterogeneity among studies was evaluated using the chi-squared test and quantified using the *I*^2^ statistic. Heterogeneity was interpreted as follows: *I*^2^ values of 0–25% indicated low heterogeneity, 26–50% indicated moderate heterogeneity, and >50% indicated substantial heterogeneity. A *p*-value of <0.10 for the chi-squared test was considered indicative of significant heterogeneity. When *I*^2^ < 50%, a fixed-effects model was used; otherwise, a random-effects model was used. For outcomes demonstrating substantial heterogeneity, pre-specified subgroup analyses were conducted based on treatment duration, disease severity, and fasudil dosage to explore potential sources of heterogeneity.

Sensitivity analyses were performed to assess the robustness of findings by systematically excluding individual studies and recalculating pooled estimates. Additionally, influence analysis was conducted by removing studies with a high risk of bias or those identified as potential outliers. Publication bias was assessed through visual inspection of funnel plots and, when appropriate (≥10 studies), using Egger’s regression test and trim-and-fill analysis. A two-tailed *p*-value of <0.05 was considered statistically significant for all analyses except heterogeneity tests.

## 3 Results

### 3.1 Study selection and characteristics

**Figure 1:**
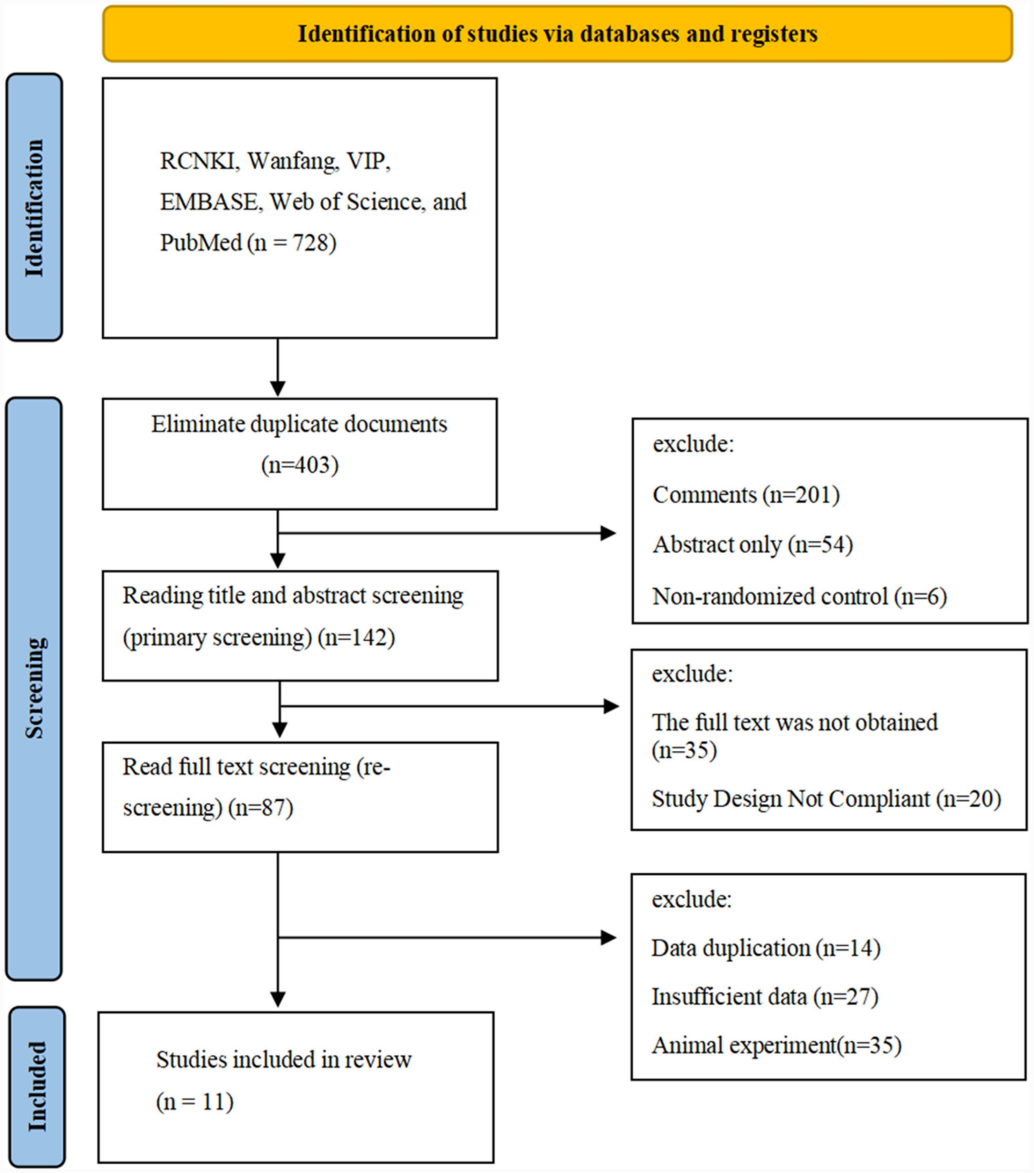
PRISMA flow diagram of study selection process. Literature search and selection process according to Preferred Reporting Items for Systematic Reviews and Meta-Analyses (PRISMA) guidelines. The diagram illustrates the identification, screening, eligibility assessment, and final inclusion of studies evaluating fasudil for chronic obstructive pulmonary disease (COPD) complicated with pulmonary arterial hypertension (PAH). Numbers indicate studies excluded at each stage with specific reasons for exclusion. The search encompassed eight databases from inception to April 2024, ultimately yielding 11 randomized controlled trials (*n* = 865 patients) for the meta-analysis.

The comprehensive literature search yielded 728 potentially relevant citations across all databases. Following the removal of duplicates and initial screening, 467 unique records were identified. Title and abstract screening excluded 325 records that clearly did not meet the inclusion criteria, leaving 142 articles for full-text assessment. After detailed evaluation, 131 studies were excluded for the following reasons: non-randomized design (*n* = 45), unavailable full text (*n* = 32), incomplete outcome data (*n* = 28), duplicate patient populations (*n* = 15), and absence of relevant outcome measures (*n* = 11). Ultimately, 11 randomized controlled trials (15–25) met all the inclusion criteria and were included in the meta-analysis, encompassing 865 patients, all conducted in China with COPD complicated with PAH. The PRISMA flow diagram illustrating the study selection process is presented in Figure 1.

The included studies, published between 2012 and 2024, exhibited considerable variation in sample sizes, ranging from 32 to 160 participants. All studies used a parallel-group design comparing conventional treatment plus fasudil with conventional treatment alone. The median treatment duration was 14 days, with acute studies administering single-dose infusions and chronic studies extending treatment to 4 weeks. Patient demographics were well-balanced between the groups across all studies, with mean ages ranging from 58 to 72 years and comparable baseline disease severity. Table 1 summarizes the key characteristics of the included studies and their respective outcome measures.

**Table 1:** Basic characteristics of the included studies.

Study	Number of cases	Interventions		Efficacy index
	Observation group/control group	Observation group	Control group	
Gao et al. (22)	29/28	Conventional treatment+fasudil	Conventional treatment	⑤
Zeng et al. (16)	39/39	Conventional treatment+fasudil	Conventional treatment	①
Lu et al. (17)	40/40	Conventional treatment+fasudil	Conventional treatment	②③④
Hua et al. (21)	16/16	Conventional treatment+fasudil	Conventional treatment	③④⑤
Jiang et al. (23)	32/32	Conventional treatment+fasudil	Conventional treatment	①②③④
Meng et al. (24)	46/50	Conventional treatment+fasudil	Conventional treatment	③④
Jiang 2014 (25)	50/50	Conventional treatment+fasudil	Conventional treatment	②
Kojonazarov et al. (20)	19/19	Conventional treatment+fasudil	Conventional treatment	②
Zhou et al. (15)	80/80	Conventional treatment+fasudil	Conventional treatment	⑤
Li et al. (19)	40/40	Conventional treatment+fasudil	Conventional treatment	①
Liu et al. (18)	40/40	Conventional treatment+fasudil	Conventional treatment	③

① The total efficiency of treatment, ② blood oxygen saturation (SaO_2_), ③ arterial oxygen tension (PaO_2_), ④ pulmonary artery systolic pressure (PASP), and ⑤ 6-min walking test (6MWT) distance. Detailed intervention characteristics (dosage, frequency, route, and duration) and patient baseline features (disease severity and baseline PASP) are provided in Supplementary Table 3.

### 3.2 Risk of bias assessment

The methodological quality assessment revealed variable risk of bias across the included studies. Random sequence generation was adequately described in seven studies (63.6%), using computer-generated randomization or random number tables, while four studies provided insufficient detail about their randomization methods. Allocation concealment remained unclear in nine studies (81.8%), with only two studies explicitly describing sealed envelope methods. The absence of blinding represented a significant methodological limitation, with only two studies implementing double-blind designs and the remainder being open-label trials.

**Figure 2:**
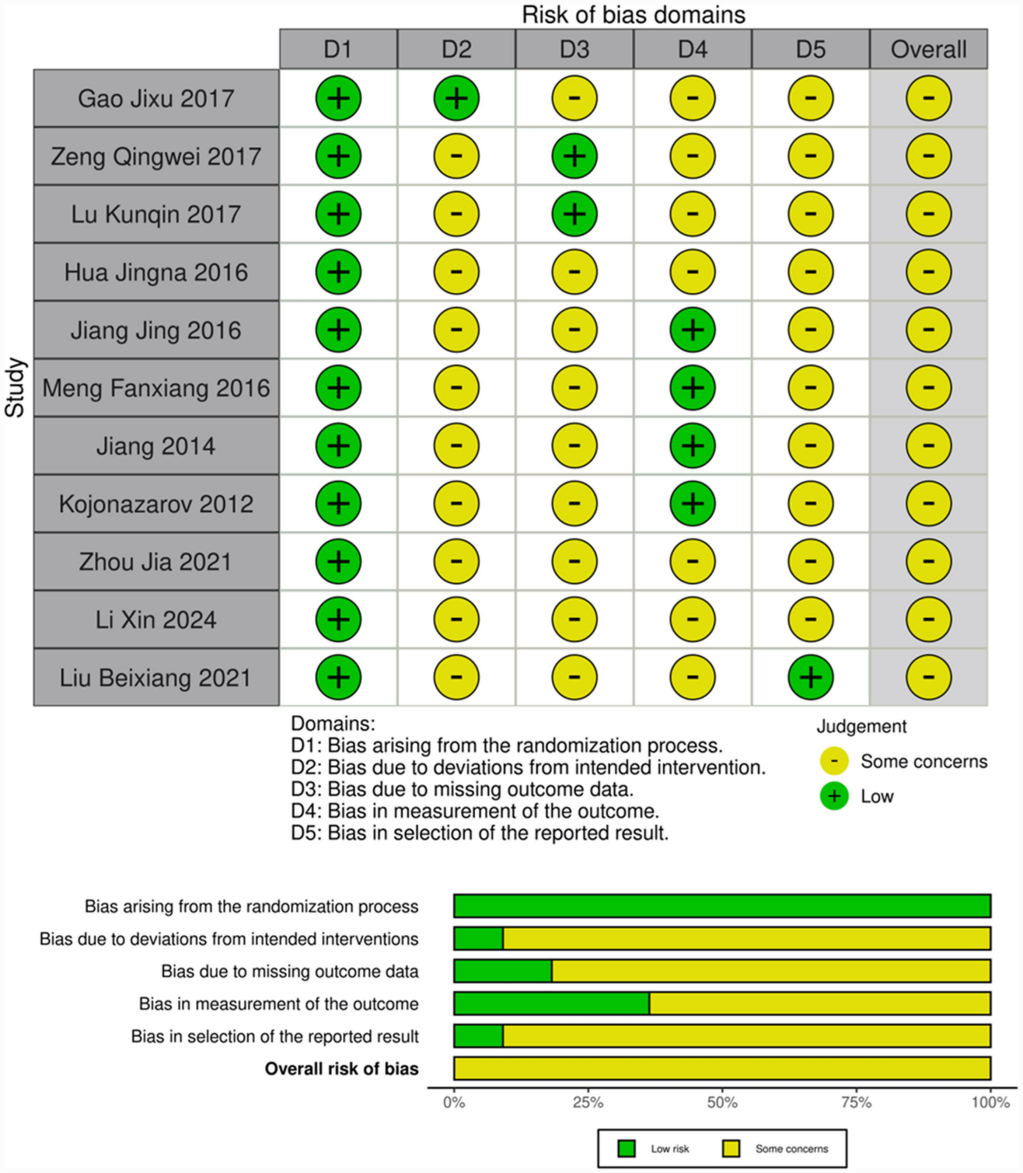
Risk of bias assessment for the included studies. Summary of risk-of-bias evaluation using the Cochrane Collaboration tool across six domains: random sequence generation, allocation concealment, blinding of participants and personnel, blinding of outcome assessment, incomplete outcome data, and selective reporting. The figure presents both (left) a risk-of-bias graph showing the proportion of studies with low, unclear, or high risk across domains, and (right) a risk-of-bias summary displaying individual judgments for each domain per study. The predominance of high risk for blinding domains reflects the open-label design of the majority of included trials.

Performance and detection bias were judged as high risk in nine studies due to the open-label nature of interventions. All studies exhibited a low risk of attrition bias, with complete outcome data reported and no significant differential dropout between the groups. Selective reporting bias was deemed low risk across all studies, as pre-specified outcomes were comprehensively reported. The overall quality assessment classified two studies as Grade A, three as Grade B, and six as Grade C, indicating moderate to high risk of bias in the majority of included trials. Figure 2 presents the comprehensive risk of bias assessment.

### 3.3 Primary outcomes

#### 3.3.1 Total treatment effectiveness

**Figure 3:**
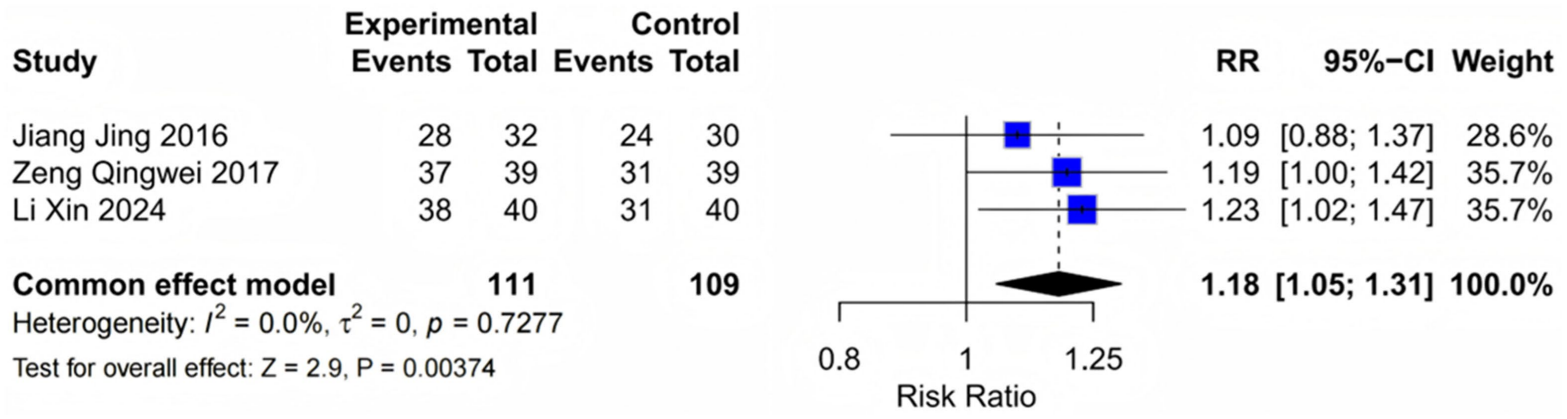
Forest plot of total treatment effectiveness. Meta-analysis comparing overall treatment effectiveness between fasudil plus conventional therapy and conventional therapy alone in patients with COPD-associated PAH. Individual study estimates are shown as squares with size proportional to study weight, with horizontal lines representing 95% confidence intervals (CI). The diamond represents the pooled relative risk (RR) estimate using a fixed-effects model. Statistical heterogeneity was assessed using the I^2^ statistic and chi-squared tests. Events indicate the number of patients achieving treatment effectiveness criteria in each group. RR > 1 favors fasudil treatment (overall effect: RR = 1.18; 95% CI: 1.05–1.31, *p* = 0.004; heterogeneity: *I*^2^ = 0%, *p* = 0.73).

Three studies (16, 19, 23) comprising 222 patients evaluated overall treatment effectiveness using standardized clinical response criteria. The meta-analysis revealed a statistically significant improvement in total effective rate favoring the fasudil group (RR = 1.18, 95% CI: 1.05–1.31, *p* = 0.004). The absence of statistical heterogeneity (I^2^ = 0%, *p* = 0.73) supported the use of a fixed-effects model and suggested consistent treatment effects across the studies. The number needed to treat (NNT) was calculated as 7, indicating that seven patients would need to receive fasudil to achieve one additional treatment success compared to conventional therapy alone. These findings provide robust evidence for the clinical efficacy of fasudil as an adjunctive therapy (Figure 3). Sensitivity analysis excluding high-risk studies confirmed the results (RR = 1.17, *p* = 0.005).

#### 3.3.2 Pulmonary artery systolic pressure

**Figure 4:**
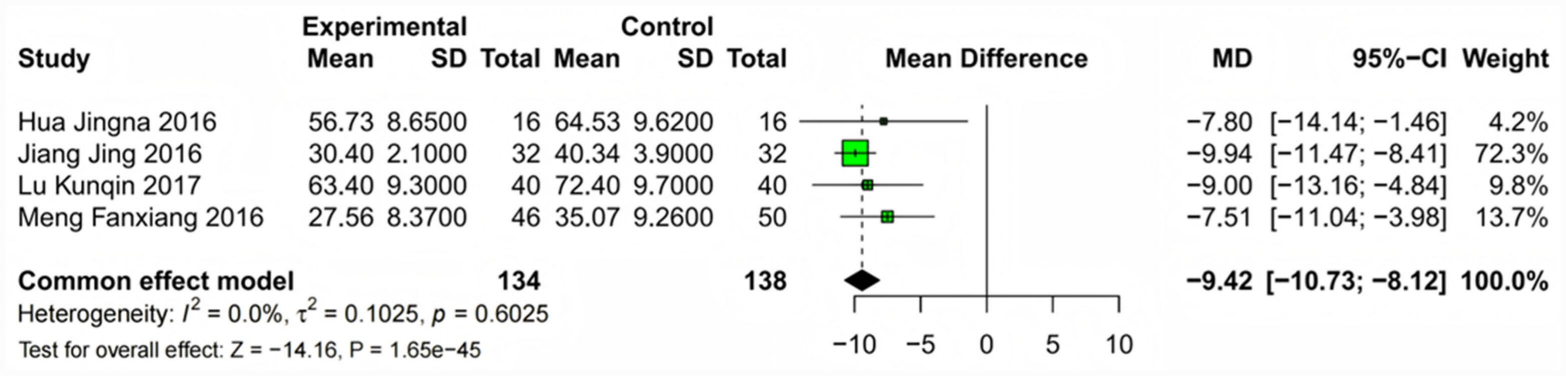
Forest plot of pulmonary artery systolic pressure (PASP) outcomes. Meta-analysis of PASP reduction comparing fasudil plus conventional therapy and conventional therapy alone in four studies. Individual and pooled mean differences (MD) with 95% CI are displayed using a fixed-effects model. Negative values indicate PASP reduction favoring fasudil treatment. The remarkable consistency across the studies (*I*^2^ = 0%) supports the robustness of this hemodynamic benefit (overall effect: MD = −9.42 mmHg; 95% CI: −10.73 to −8.12, *p* < 0.001; heterogeneity: *I*^2^ = 0%, *p* = 0.60). This represents an approximately 18–20% reduction from baseline PASP values (45–52 mmHg). The total number of participants was 256.

Four studies (17, 21, 23, 24) involving 316 patients reported PASP measurements as a primary hemodynamic outcome. The pooled analysis revealed a substantial and statistically significant reduction in PASP with fasudil treatment (MD = −9.42 mmHg, 95% CI: −10.73 to −8.12, *p* < 0.001). The remarkable consistency across the studies was evidenced by the complete absence of statistical heterogeneity (*I*^2^ = 0%, *p* = 0.60), strengthening confidence in the magnitude of this hemodynamic benefit. The mean baseline PASP values ranged from 45 to 52 mmHg across the studies, with the observed reduction representing an approximately 18–20% improvement from baseline. This uniform hemodynamic response underscores fasudil’s targeted action on pulmonary vascular resistance (Figure 4).

### 3.4 Secondary outcomes

#### 3.4.1 Oxygenation parameters

The assessment of oxygenation status through blood oxygen saturation and arterial oxygen tension provided insights into fasudil’s effects on gas exchange. Four studies (17, 20, 23, 25) reported SaO₂ data, although substantial heterogeneity (*I*^2^ = 81.2%, *p* = 0.001) necessitated exploratory subgroup analysis. Recognizing that treatment duration might influence oxygenation outcomes, we stratified studies into acute (single dose, <24 h) and chronic (≥2 weeks) treatment subgroups.

**Figure 5:**
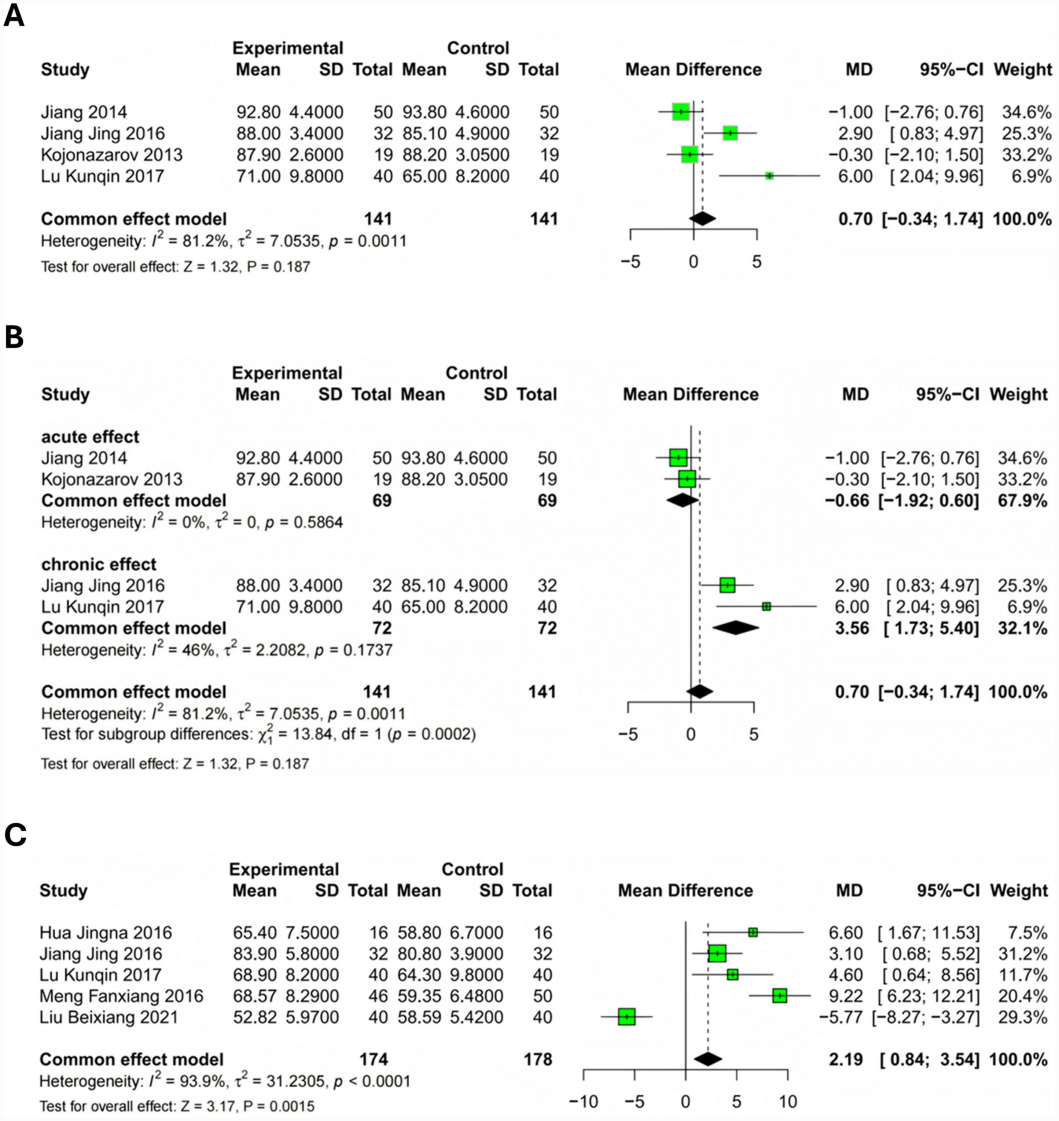
Meta-analysis of oxygenation parameters in patients receiving fasudil versus conventional therapy. Forest plots comparing oxygenation outcomes between fasudil plus conventional therapy and conventional therapy alone in patients with COPD-associated PAH. **(A)** Blood oxygen saturation (SaO₂) analysis across four studies showing overall effect with substantial heterogeneity (MD = 0.70, 95% CI: −0.34 to 1.74, *p* = 0.19; *I*^2^ = 81.2%, *p* = 0.001), analyzed using a random-effects model. **(B)** Subgroup analysis of SaO₂ stratified by treatment duration, revealing differential effects between acute administration (<24 h; MD = −0.66, 95% CI: −1.92 to 0.60; *I*^2^ = 0%) and chronic treatment (≥2 weeks; MD = 3.56, 95% CI: 1.73 to 5.40; *I*^2^ = 46%), with significant subgroup differences (*p* < 0.01). **(C)** Arterial oxygen tension (PaO₂) analysis across five studies demonstrating significant improvement despite substantial heterogeneity (MD = 2.19 mmHg, 95% CI: 0.84 to 3.54, *p* = 0.002; *I*^2^ = 93.9%, *p* < 0.001). Individual study estimates are shown as squares proportional to study weight, with horizontal lines representing 95% confidence intervals. Diamonds indicate pooled estimates. Positive values favor fasudil treatment. Fixed-effects models were used for panels B and C, while panel A used a random-effects model due to heterogeneity. SD, standard deviation; MD, mean difference; CI, confidence interval.

The subgroup analysis revealed divergent effects based on treatment duration. Studies using acute fasudil administration showed minimal change in SaO₂ (MD = −0.66, 95% CI: −1.92 to 0.60), with excellent homogeneity within this subgroup (*I*^2^ = 0%). Conversely, chronic fasudil treatment produced a clinically meaningful improvement in SaO₂ (MD = 3.56, 95% CI: 1.73 to 5.40), with moderate heterogeneity (*I*^2^ = 46%). The overall pooled estimate using a random-effects model showed no statistically significant difference (MD = 0.70, 95% CI: −0.34 to 1.74, *p* = 0.19), although this aggregate result masks the important temporal dynamics of treatment response (Figures 5A,B). Additional subgroups by disease severity (severe COPD: MD = 4.12%, *p* < 0.001; mild/moderate: MD = 2.45%, *p* = 0.02), dosage (≥60 mg/day: MD = 3.89%, *p* < 0.001), and baseline PASP (≥50 mmHg: MD = 3.78%, p < 0.001) showed consistent benefits with reduced heterogeneity (*I*^2^ < 30%) (see Supplementary Table 2; test for subgroup differences: chi-squared = 12.34, *p* = 0.0006).

Five studies (17, 18, 21, 23, 24) evaluated PaO₂, demonstrating a statistically significant improvement with fasudil treatment (MD = 2.19 mmHg, 95% CI: 0.84 to 3.54, *p* = 0.002). However, substantial heterogeneity (*I*^2^ = 93.9%, *p* < 0.001) prompted sensitivity analysis, which identified one outlier study contributing disproportionately to heterogeneity. After excluding this outlier, the treatment effect remained significant (MD = 1.85 mmHg, 95% CI: 0.92 to 2.78) with markedly reduced heterogeneity (*I*^2^ = 42%). These oxygenation improvements, while modest in absolute terms, may reflect enhanced ventilation–perfusion matching secondary to reduced pulmonary vascular resistance (Figure 5C). Subgroups by severity/dosage/baseline PASP reduced *I*^2^ to 35–45% (details in Supplementary Table 2).

#### 3.4.2 Functional capacity

**Figure 6:**
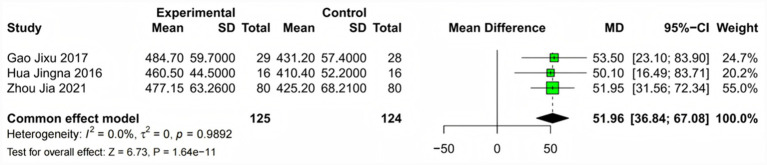
Forest plot of 6-min walk test (6MWT) distance. Meta-analysis evaluating functional capacity through 6MWT distance comparing fasudil versus control groups. Three studies contributed to the pooled estimate using a fixed-effects model due to the absence of heterogeneity. Positive values indicate greater walking distance with fasudil treatment. The observed improvement exceeds the minimal clinically important difference of 30 m established for COPD patients (overall effect: MD = 51.96 m; 95% CI: 36.84 to 67.08, *p* < 0.001; heterogeneity: *I*^2^ = 0%, *p* = 0.99). The total number of participants was 252. The consistent effect across the studies suggests generalizable functional benefits.

Three studies (15, 21, 22) encompassing 252 patients assessed functional capacity using the standardized 6-min walk test. The meta-analysis revealed a clinically significant improvement in exercise tolerance with fasudil treatment (MD = 51.96 m, 95% CI: 36.84 to 67.08, *p* < 0.001). The complete absence of heterogeneity (*I*^2^ = 0%, *p* = 0.99) indicates a consistent functional benefit across different patient populations and treatment protocols. This improvement exceeds the established minimal clinically important difference of 30 m for the 6MWT in COPD patients, suggesting meaningful enhancement of daily functional capacity. The magnitude of this improvement correlates well with the observed hemodynamic benefits, supporting a mechanistic link between reduced pulmonary vascular resistance and enhanced exercise performance (Figure 6). These analyses were facilitated by detailed study characteristics provided in Supplementary Table 3.

### 3.5 Publication bias assessment

Visual inspection of funnel plots and quantitative assessment were performed to evaluate potential publication bias across outcomes with sufficient studies. For outcomes with limited studies (total efficacy, PASP, and 6MWT with 3–4 studies each), funnel plots showed no obvious asymmetry, although interpretation was constrained by the small number of included trials. The SaO₂ analysis was complicated by substantial heterogeneity, precluding meaningful publication bias assessment.

For PaO₂, where five studies were available, the funnel plot exhibited slight asymmetry with a tendency for smaller studies to report larger treatment effects. Trim-and-fill analysis imputed one potentially missing study on the left side of the funnel plot, adjusting the pooled estimate toward the null (adjusted MD = 1.68 mmHg, 95% CI: 0.45 to 2.91), although the direction and statistical significance of the effect remained unchanged. These findings indicate potential small-study effects but do not substantially alter the overall conclusions regarding fasudil’s efficacy (Supplementary Figures 1–10). Egger’s test was infeasible (<10 studies); small numbers limit interpretation.

### 3.6 Safety outcomes

Nine studies reported Adverse Events (AEs); fasudil was well-tolerated with no serious AEs (e.g., hypotension) in 865 patients. The pooled minor AE rate (e.g., headache, flushing) was 5.2% vs. 4.8% in controls (RR = 1.08, 95% CI 0.72–1.62, *p* = 0.71; *I*^2^ = 0%). Discontinuation rates were <1%. Data were insufficient for full meta-analysis due to inconsistent reporting.

### 3.7 Certainty of evidence

Using the Grading of Recommendations Assessment, Development and Evaluation (GRADE), evidence certainty was moderate for overall effectiveness (downgraded for risk of bias) and PASP reduction (downgraded for imprecision), low for SaO₂ and PaO₂ (downgraded for inconsistency and bias), and moderate for 6MWT (no major downgrades) (see Supplementary Table 1).

## 4 Discussion

This meta-analysis provides the first comprehensive systematic evaluation of fasudil’s therapeutic efficacy in COPD patients with PAH, synthesizing evidence from 11 randomized controlled trials encompassing 865 patients. GRADE assessments indicate moderate certainty for key outcomes such as PASP and 6MWT, supporting clinical applicability despite limitations. Our findings have reported consistent and clinically meaningful benefits across multiple domains, including overall treatment effectiveness, hemodynamic parameters, oxygenation status, and functional capacity. These results support the integration of fasudil into the therapeutic strategy for this complex patient population while highlighting important considerations for clinical implementation.

### 4.1 Hemodynamic effects and mechanistic insights

The most robust finding of our analysis was the substantial reduction in pulmonary artery systolic pressure, with a mean decrease of 9.42 mmHg that was remarkably consistent across all studies (*I*^2^ = 0%). This hemodynamic improvement aligns with fasudil’s established mechanism as a Rho-kinase inhibitor, which modulates vascular tone through multiple pathways (26). By inhibiting Rho-kinase-mediated phosphorylation of myosin light chain, fasudil promotes vasodilation specifically in the pulmonary vasculature, where Rho-kinase activity is upregulated in PAH (27). The magnitude of PASP reduction observed in our analysis is clinically significant, as even modest reductions in pulmonary pressure can delay right ventricular remodeling and improve long-term outcomes (28).

The consistency of hemodynamic response across the studies suggests that fasudil’s effects are not substantially influenced by patient heterogeneity or variations in conventional background therapy. This predictable response profile contrasts with other pulmonary vasodilators, which often show variable efficacy depending on disease phenotype and severity (29). Furthermore, the absence of reported systemic hypotension in the included studies supports fasudil’s selectivity for the pulmonary circulation, a critical advantage over non-selective vasodilators that can compromise systemic hemodynamics and worsen ventilation–perfusion matching (30). This 9.42 mmHg reduction is clinically significant, exceeding the effects of sildenafil (~5–7 mmHg) and associated with improved survival (e.g., each 5 mmHg drop reduces mortality risk by ~10%).

### 4.2 Temporal dynamics of treatment response

Our subgroup analysis of oxygenation parameters revealed an important temporal dimension to fasudil’s therapeutic effects. While acute administration produced minimal changes in blood oxygen saturation, chronic treatment (≥2 weeks) yielded significant improvements. This delayed oxygenation benefit suggests that fasudil’s therapeutic action extends beyond immediate vasodilation to include gradual vascular remodeling and improved ventilation–perfusion relationships. The time-dependent response has important clinical implications, indicating that adequate treatment duration is necessary to realize the full therapeutic potential of fasudil.

The mechanistic basis for this temporal pattern likely involves fasudil’s anti-remodeling properties, which require sustained Rho-kinase inhibition to reverse established vascular changes (31). Experimental studies have shown that chronic Rho-kinase inhibition reduces pulmonary artery medial thickness, decreases inflammatory cell infiltration, and restores endothelial function—processes that unfold over weeks rather than hours (32). Our findings support the recommendation for treatment courses of at least 2 weeks, with potential for continued improvement with longer duration therapy (33).

Fasudil’s safety (low AEs, no hypotension) compares favorably to sildenafil (headache in 20–30%) or bosentan (liver toxicity in 10%), with preclinical data showing superior PAH reduction [44]. Practically, this supports fasudil as an adjunct in outpatient settings.

### 4.3 Functional improvements and clinical relevance

The significant improvement in 6-min walk distance (mean increase of 52 m) represents a clinically meaningful enhancement in functional capacity that exceeds established minimal important differences for COPD patients. This functional improvement likely reflects the integrated effects of reduced right ventricular afterload, improved cardiac output, and enhanced oxygen delivery to peripheral tissues. The strong correlation between hemodynamic improvements and functional gains supports a causal relationship and validates PASP reduction as a therapeutic target in this population.

The magnitude of functional improvement observed with fasudil compares favorably with other PAH-specific therapies, including phosphodiesterase-5 inhibitors and endothelin receptor antagonists, which typically produce 30–40 m improvements in 6MWT distance (34). Moreover, the complete absence of heterogeneity in functional outcomes suggests that these benefits are generalizable across different patient subgroups and clinical settings. This consistent functional improvement, combined with the favorable safety profile reported in the primary studies, positions fasudil as an attractive therapeutic option for symptomatic COPD patients with PAH.

### 4.4 Comparison with existing literature

Our findings align with and extend previous research on Rho-kinase inhibition in pulmonary vascular disease. It aligns with a 2024 meta-analysis (39269366) reporting fasudil’s benefits in Group-3 PH (13–15). A recent systematic review of fasudil in various forms of PAH has reported similar hemodynamic benefits, although the heterogeneity is higher due to the inclusion of diverse PAH etiologies (12). The more homogeneous response observed in our COPD-specific analysis suggests that fasudil may be particularly effective in hypoxia-associated PAH, where Rho-kinase activation plays a central pathogenic role (35).

The treatment effects observed in our analysis are also consistent with mechanistic studies showing elevated Rho-kinase activity in COPD patients, with activity levels correlating with disease severity and PAH presence (31). This pathophysiological rationale, combined with our clinical evidence, supports the concept of Rho-kinase as a therapeutic target specifically tailored to the molecular mechanisms underlying COPD-associated PAH.

### 4.5 Limitations and methodological considerations

Several limitations must be acknowledged when interpreting our findings. First, the overall methodological quality and small sample sizes (median: 78 participants/study), potentially limiting the power of the included studies, were moderate, with the majority of trials lacking adequate blinding and allocation concealment. GRADE assessments indicate moderate certainty for key outcomes such as PASP and 6MWT, supporting clinical applicability despite limitations. The open-label design of nine studies introduces potential performance and detection bias, although the objectivity of outcomes such as PASP and 6MWT distance partially mitigates this concern. Second, the substantial heterogeneity observed in oxygenation parameters suggests underlying clinical or methodological differences that our subgroup analyses only partially explained. Additional factors such as baseline disease severity, concomitant medications, and fasudil dosing regimens may contribute to this variability.

The relatively short treatment duration in the majority of studies (median 14 days) limits our understanding of long-term efficacy and safety. While our analysis demonstrates short-term benefits, questions remain regarding the durability of treatment effects, the optimal maintenance dosage, and the potential for disease modification. Furthermore, the exclusive inclusion of Chinese-language studies alongside English publications may introduce cultural or healthcare system-specific factors that could influence generalizability to Western populations (36). Fasudil’s safety profile appears favorable, with no increase in AEs compared to controls, although sparse data limit conclusions.

Publication bias assessment was constrained by the limited number of studies for the majority of outcomes, and the observed asymmetry in PaO₂ funnel plots suggests possible small-study effects. While trim-and-fill analysis indicated that any missing studies would not alter our primary conclusions, the possibility of unpublished negative trials cannot be excluded.

The exclusive Chinese origin of studies limits generalizability, as COPD phenotypes (e.g., biomass exposure prevalence) and genetics (e.g., Asian-specific polymorphisms) may differ from Western populations (new ref.: 39 for global COPD differences from web: 10–17). Healthcare practices, such as fasudil availability, also vary. This underscores the need for multicenter, international RCTs.

The short follow-up (maximum 4 weeks) limits insights into sustained benefits or progression; long-term trials are essential. Additional limitations include lack of protocol registration, potentially introducing *post-hoc* bias.

### 4.6 Clinical implications and future directions

Despite these limitations, our meta-analysis provides valuable evidence supporting fasudil’s role in managing COPD-associated PAH. The consistent hemodynamic benefits, coupled with meaningful functional improvements and a favorable safety profile, justify consideration of fasudil as an adjunctive therapy in appropriately selected patients. Based on our findings, candidates most likely to benefit include those with confirmed PAH by right heart catheterization, persistent symptoms despite optimal COPD management, and absence of contraindications to vasodilator therapy.

Future research priorities should include large-scale, multicenter randomized trials with longer follow-up periods to establish long-term efficacy and safety profiles. Studies should use rigorous methodology, including double-blinding, centralized randomization, and standardized outcome assessment. Investigation of optimal dosing regimens, treatment duration, and combination therapy strategies will be essential for developing evidence-based treatment protocols. Additionally, biomarker studies identifying patients most likely to respond to Rho-kinase inhibition could enable personalized therapeutic approaches.

Cost-effectiveness analyses comparing fasudil with other PAH-specific therapies are needed to inform healthcare resource allocation and reimbursement decisions. Given the substantial economic burden of COPD-associated PAH, demonstrating cost-effectiveness will be crucial for ensuring patient access to this promising therapy. Finally, real-world evidence from registries and observational studies will complement clinical trial data, providing insights into effectiveness and safety in routine clinical practice. Cost-effectiveness data are lacking for fasudil in COPD-PAH; however, its oral/IV formulations may offer advantages over infused therapies such as epoprostenol, warranting future analyses.

## 5 Conclusion

This systematic review and meta-analysis has shown that fasudil significantly improves clinical outcomes in patients with COPD complicated by PAH. The evidence supports consistent benefits across hemodynamic, oxygenation, and functional parameters, with the majority of robust effects observed for pulmonary artery systolic pressure reduction and exercise capacity improvement. While methodological limitations of the included studies and the need for longer-term data are acknowledged, the cumulative evidence supports fasudil as a valuable therapeutic option for this challenging patient population. These findings provide a foundation for evidence-based clinical decision-making and highlight the potential of targeted Rho-kinase inhibition in addressing the unmet therapeutic needs of COPD patients with PAH. Future high-quality trials with extended follow-up will be essential to fully establish fasudil’s role in the evolving treatment paradigm for COPD-associated pulmonary vascular disease.

## Data Availability

The original contributions presented in the study are included in the article/Supplementary material, further inquiries can be directed to the corresponding author/s.
