# Supplementary material for: Efficacy of fasudil in COPD-associated pulmonary arterial hypertension: meta-analysis of randomized controlled trials
Source: Front Med (Lausanne). 2026 Jan 26;13:1723597. doi: 10.3389/fmed.2026.1723597 (PMC12883821; doi:10.3389/fmed.2026.1723597)
Supplement: Supplementary file 1 [file Table_1.docx]

**Supplementary Table 1:** GRADE Summary of Findings

| Outcome | Anticipated Effects (95% CI) | Relative Effect (95% CI) | No. of Participants (Studies) | Certainty of Evidence (GRADE) | Reasons for Downgrade |
| --- | --- | --- | --- | --- | --- |
| Overall Treatment Effectiveness | Risk with fasudil: 85% effective vs. 72% control (NNT=7) | RR 1.18 (1.05 to 1.31) | 222 (3 RCTs) | Moderate (⊕⊕⊕◯) | Downgraded 1 level for risk of bias (open-label in most studies); no inconsistency (I²=0%). |
| Pulmonary Artery Systolic Pressure (PASP) | Mean reduction: -9.42 mmHg lower with fasudil | MD -9.42 (-10.73 to -8.12) | 316 (4 RCTs) | Moderate (⊕⊕⊕◯) | Downgraded 1 level for imprecision (small total sample) and risk of bias; no inconsistency (I²=0%). |
| Blood Oxygen Saturation (SaO₂) - Overall | Mean increase: 0.70% higher with fasudil (not significant) | MD 0.70 (-0.34 to 1.74) | 320 (4 RCTs, estimated from text) | Low (⊕⊕◯◯) | Downgraded 2 levels for inconsistency (I²=81%) and risk of bias. |
| SaO₂ - Chronic Subgroup (≥2 weeks) | Mean increase: 3.56% higher with fasudil | MD 3.56 (1.73 to 5.40) | ~200 (2 RCTs) | Moderate (⊕⊕⊕◯) | Downgraded 1 level for risk of bias; moderate inconsistency (I²=46%). |
| Arterial Oxygen Tension (PaO₂) | Mean increase: 2.19 mmHg higher with fasudil | MD 2.19 (0.84 to 3.54) | 400 (5 RCTs, estimated) | Low (⊕⊕◯◯) | Downgraded 2 levels for inconsistency (I²=94%) and publication bias (funnel asymmetry). |
| 6-Minute Walk Test (6MWT) Distance | Mean increase: 51.96 meters greater with fasudil (exceeds MCID of 30m) | MD 51.96 (36.84 to 67.08) | 252 (3 RCTs) | Moderate (⊕⊕⊕◯) | Downgraded 1 level for risk of bias; no inconsistency (I²=0%). |
| Adverse Events (Safety) | Minor AEs: 5.2% with fasudil vs. 4.8% control (no serious AEs reported) | RR 1.08 (0.72 to 1.62) | 865 (9 RCTs reporting) | Moderate (⊕⊕⊕◯) | Downgraded 1 level for imprecision (inconsistent reporting); no inconsistency (I²=0%). |

**Note:** This table assesses the certainty of evidence for primary and secondary outcomes based on the GRADE methodology. Certainty starts as "High" for RCTs and is downgraded for study limitations (risk of bias, e.g., open-label designs), inconsistency (heterogeneity I² >50%), indirectness (none applicable), imprecision (wide CIs or small samples), and publication bias (funnel asymmetry or small-study effects). Ratings: High (⊕⊕⊕⊕), Moderate (⊕⊕⊕◯), Low (⊕⊕◯◯), Very Low (⊕◯◯◯). Data derived directly from the meta-analysis results (e.g., 11 RCTs, 865 participants total).
