# Supplementary material for: Efficacy of fasudil in COPD-associated pulmonary arterial hypertension: meta-analysis of randomized controlled trials
Source: Front Med (Lausanne). 2026 Jan 26;13:1723597. doi: 10.3389/fmed.2026.1723597 (PMC12883821; doi:10.3389/fmed.2026.1723597)
Supplement: Supplementary file 2 [file Table_2.docx]

**Supplementary Table 2:** Subgroup Analyses for Oxygenation Outcomes

| Outcome and Subgroup | No. of Studies | No. of Participants | Mean Difference (95% CI) | P-value | Heterogeneity (I²) |
| --- | --- | --- | --- | --- | --- |
| SaO₂ (%) |  |  |  |  |  |
| Disease Severity: Mild/Moderate COPD | 2 | ~150 | 2.45 (0.50 to 4.40) | 0.02 | 25% |
| Disease Severity: Severe COPD | 2 | ~170 | 4.12 (2.20 to 6.04) | <0.001 | 35% |
| Fasudil Dosage: <60 mg/day | 2 | ~160 | 2.80 (0.90 to 4.70) | 0.004 | 20% |
| Fasudil Dosage: ≥60 mg/day | 2 | ~160 | 3.89 (1.95 to 5.83) | <0.001 | 28% |
| Baseline PASP: <50 mmHg | 2 | ~140 | 2.60 (0.65 to 4.55) | 0.009 | 22% |
| Baseline PASP: ≥50 mmHg | 2 | ~180 | 3.78 (1.85 to 5.71) | <0.001 | 30% |
| PaO₂ (mmHg) |  |  |  |  |  |
| Disease Severity: Mild/Moderate COPD | 2 | ~180 | 1.50 (0.40 to 2.60) | 0.008 | 40% |
| Disease Severity: Severe COPD | 3 | ~220 | 2.50 (1.20 to 3.80) | <0.001 | 45% |
| Fasudil Dosage: <60 mg/day | 3 | ~200 | 1.80 (0.70 to 2.90) | 0.001 | 38% |
| Fasudil Dosage: ≥60 mg/day | 2 | ~200 | 2.40 (1.10 to 3.70) | <0.001 | 42% |
| Baseline PASP: <50 mmHg | 2 | ~170 | 1.70 (0.60 to 2.80) | 0.003 | 35% |
| Baseline PASP: ≥50 mmHg | 3 | ~230 | 2.30 (1.00 to 3.60) | <0.001 | 40% |

**Note:** This table summarizes additional pre-specified subgroup analyses for SaO₂ and PaO₂ (where heterogeneity was high), stratified by disease severity (mild/moderate vs. severe COPD, based on GOLD stages or baseline metrics in studies), fasudil dosage (<60 mg/day vs. ≥60 mg/day, common thresholds in fasudil trials), and baseline PASP (<50 mmHg vs. ≥50 mmHg, reflecting moderate vs. severe PAH). Data pooled using random-effects where I²>50%. Subgroups reduced overall heterogeneity (e.g., from 81% to <45% for SaO₂). Derived from the 4-5 studies reporting oxygenation, with consistent direction of benefit favoring fasudil.
