# Supplementary material for: Efficacy of fasudil in COPD-associated pulmonary arterial hypertension: meta-analysis of randomized controlled trials
Source: Front Med (Lausanne). 2026 Jan 26;13:1723597. doi: 10.3389/fmed.2026.1723597 (PMC12883821; doi:10.3389/fmed.2026.1723597)
Supplement: Supplementary file 3 [file Table_3.docx]

**Supplementary Table 3.** Detailed Intervention and Baseline Characteristics of Included Studie**s**

| Study (as in Table 1) | Year | Sample Size (Fasudil / Control) | Fasudil Dosage | Frequency | Route | Treatment Duration | Disease Severity | Baseline PASP (mmHg) | Outcomes Reported |
| --- | --- | --- | --- | --- | --- | --- | --- | --- | --- |
| Gao Jixu et al. | 2017 | 29 (15/14) | 30 mg | Once daily | IV infusion | 7 days | Severe COPD (GOLD III–IV) | 48 | 6MWT |
| Zeng Qingwei et al. | 2017 | 78 (39/39) | 45 mg | Once daily | IV | 14 days | Moderate–Severe COPD (GOLD II–III) | 46 | Total effectiveness |
| Lu Kunqin et al. | 2017 | 80 (40/40) | 60 mg | Once daily | IV | 7 days | Severe COPD (GOLD III–IV) | 50 | PASP, SaO₂, PaO₂ |
| Hua Jingna et al. | 2016 | 32 (16/16) | 30 mg | Once | IV | Single dose (acute) | Severe COPD (GOLD III–IV) | 48 | PaO₂, 6MWT |
| Jiang Jing et al. | 2016 | 64 (32/32) | 45 mg | Once daily | IV | 21 days | Severe COPD (GOLD III–IV) | 52 | Total effectiveness |
| Meng Fanxiang et al. | 2016 | 96 (46/50) | 45 mg | Once daily | IV | 14 days | Moderate–Severe COPD (GOLD II–III) | 47 | SaO₂, PaO₂ |
| Jiang X. et al. | 2014 | 100 (50/50) | 60 mg | Once daily | IV | 7 days | Severe COPD (GOLD III–IV) | 50 | PASP |
| Kojonazarov et al. | 2012 | 38 (19/19) | 30 mg | Once | IV | Single dose (acute) | High-altitude PAH (non-COPD) | 45 | SaO₂ |
| Zhou Jia et al. | 2021 | 160 (80/80) | 30 mg | Once daily | IV | 28 days | Severe COPD (GOLD III–IV) | 51 | PASP, PaO₂, 6MWT |
| Li Xin et al. | 2024 | 80 (40/40) | 45 mg | Once daily | IV | 14 days | Moderate COPD (GOLD II) | 46 | 6MWT |
| Liu Beixiang et al. | 2021 | 80 (40/40) | 60 mg | Once daily | IV | 21 days | Severe COPD (GOLD III–IV) | 52 |  |

**Note:** This table expands Table 1 by providing additional details on fasudil interventions (dosage, frequency, route of administration, and treatment duration) and patient baseline characteristics (disease severity assessed by GOLD stage or equivalent criteria, and baseline pulmonary artery systolic pressure). All data were extracted directly from the 11 included randomized controlled trials. Fasudil dosages and treatment durations are reported as described in the original studies, including both acute (single-dose) and short-term chronic regimens (ranging from approximately 1 to 4 weeks). One study (Kojonazarov et al., 2012) investigated high-altitude pulmonary hypertension rather than COPD-associated PAH; therefore, GOLD staging was not applicable. The total sample size across studies was 865 participants, and all trials employed a parallel-group design.
