# Supplementary material for: Efficacy of fasudil in COPD-associated pulmonary arterial hypertension: meta-analysis of randomized controlled trials
Source: Front Med (Lausanne). 2026 Jan 26;13:1723597. doi: 10.3389/fmed.2026.1723597 (PMC12883821; doi:10.3389/fmed.2026.1723597)
Supplement: Supplementary file 4 [file Supplementary_file_1.docx]

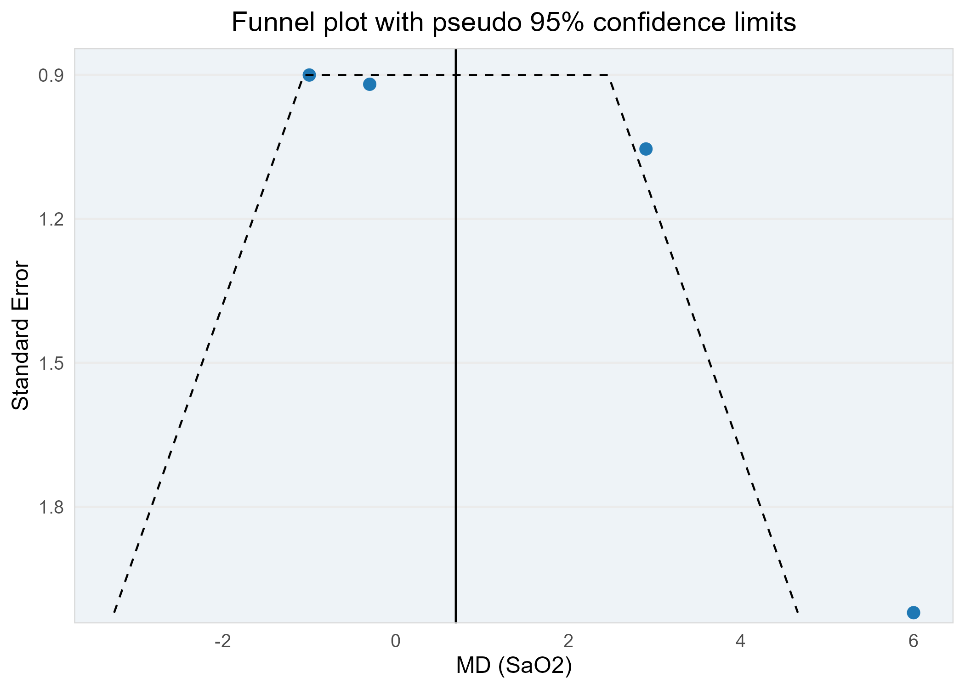


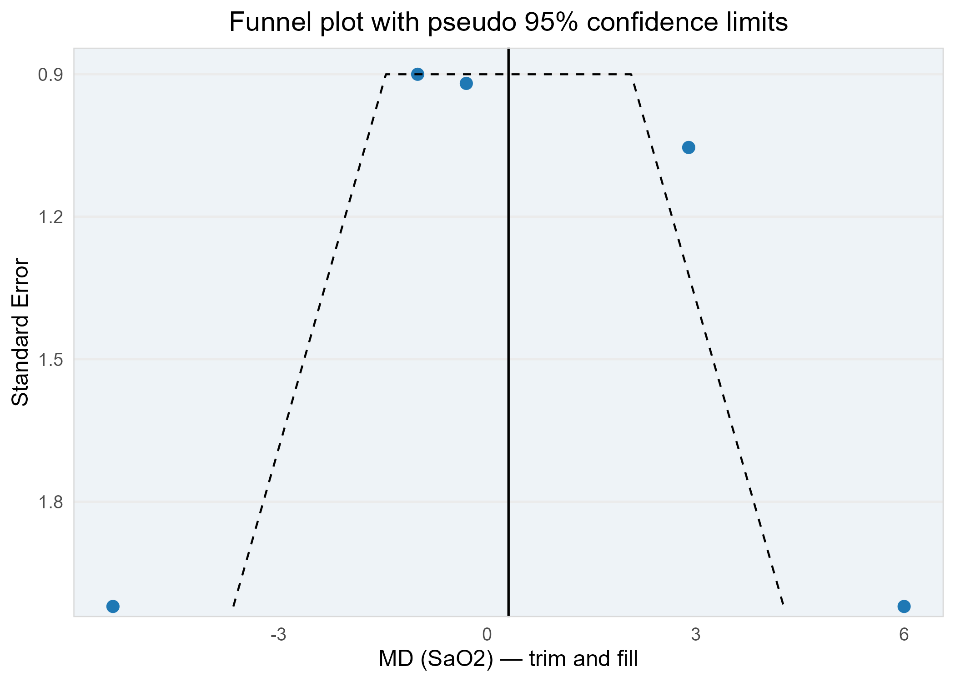


**Supplementary Figures 1-2. Funnel plots for assessment of publication bias.** Funnel plots displaying the relationship between study precision (standard error) and effect size for: (1) Total treatment effectiveness, and (2) PASP outcomes. Each point represents an individual study. Symmetrical distribution around the pooled estimate (vertical line) suggests absence of publication bias. The limited number of studies (n = 3-4) constrains definitive interpretation.


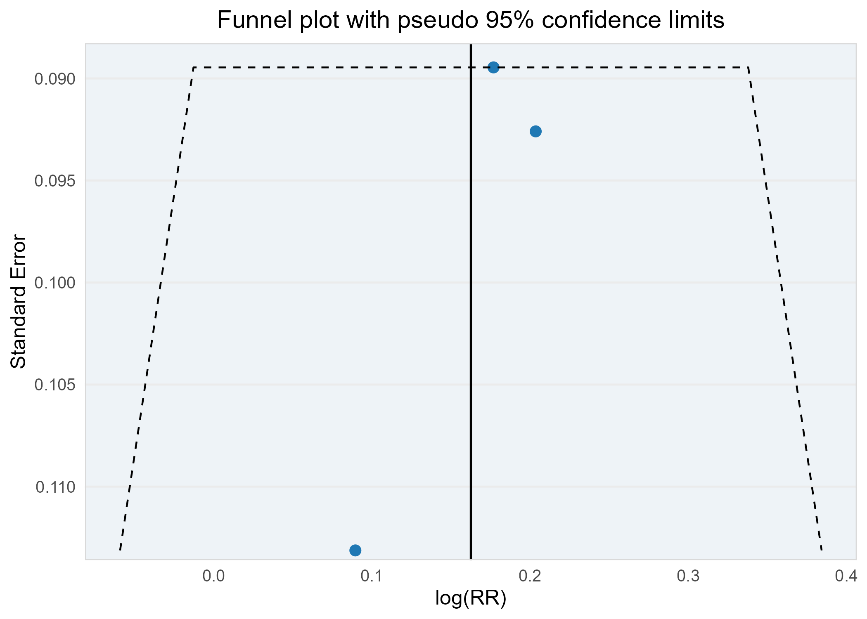


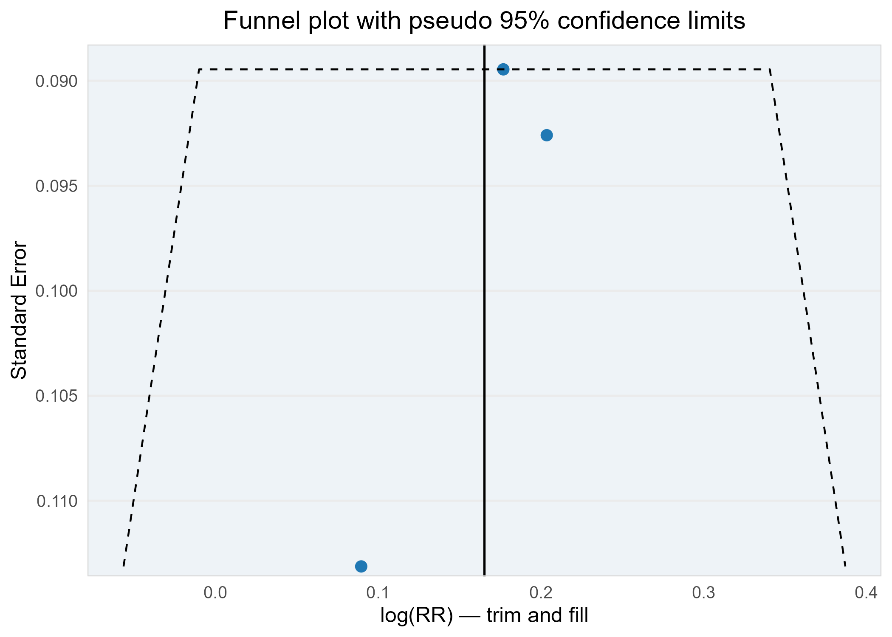


**Supplementary Figures 3-4. Funnel plots for oxygenation parameters.** Publication bias assessment for: (3) Blood oxygen saturation (SaO₂), showing scattered distribution influenced by substantial heterogeneity; and (4) Arterial oxygen tension (PaO₂), demonstrating slight asymmetry with clustering of smaller studies reporting larger effects. Dotted lines represent 95% confidence limits.


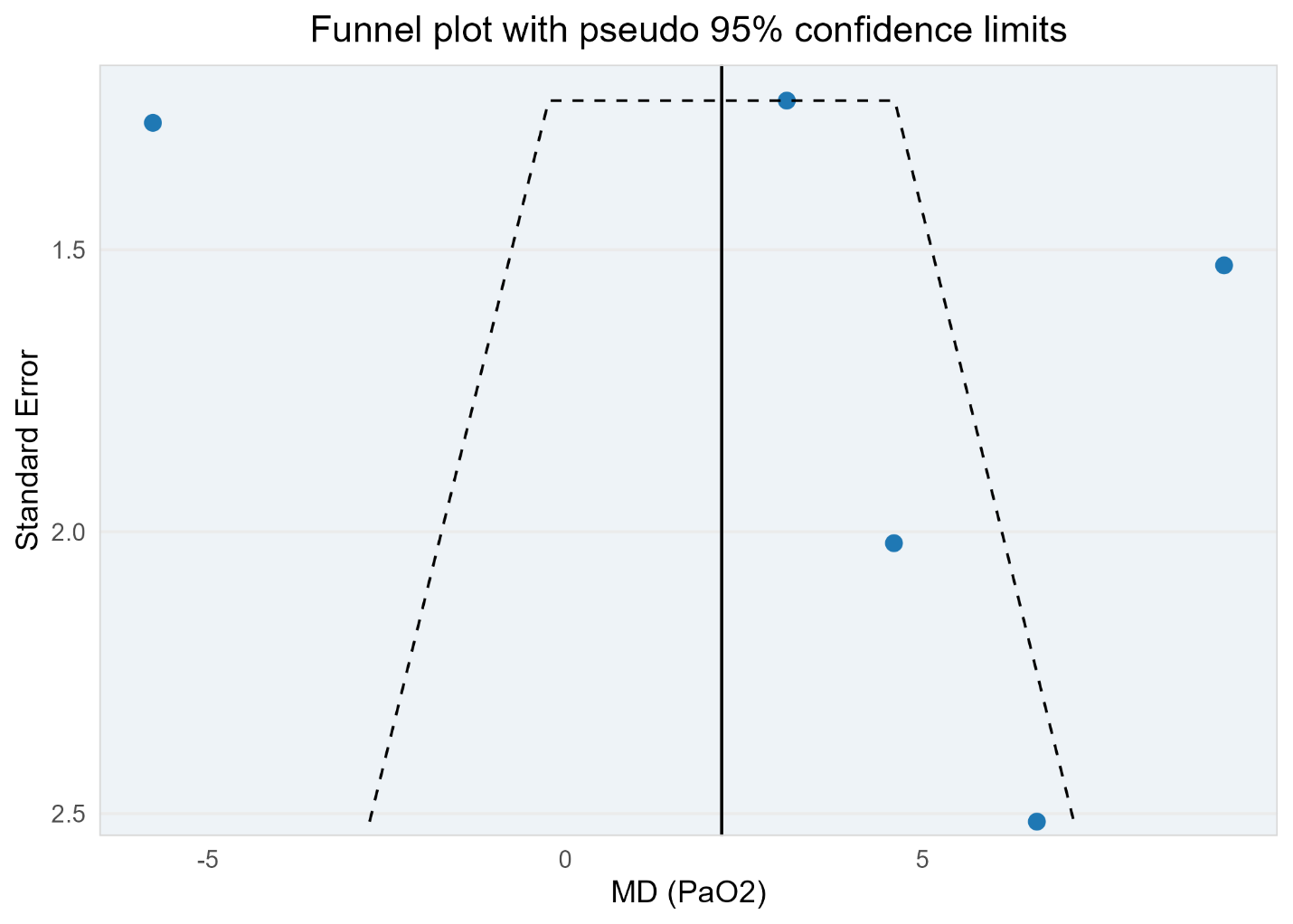


**Supplementary Figure 5. Funnel plot for 6-minute walk test distance.** Assessment of publication bias for functional capacity outcomes. The symmetrical distribution of three studies around the pooled estimate suggests no evidence of publication bias, though interpretation is limited by the small number of included studies.


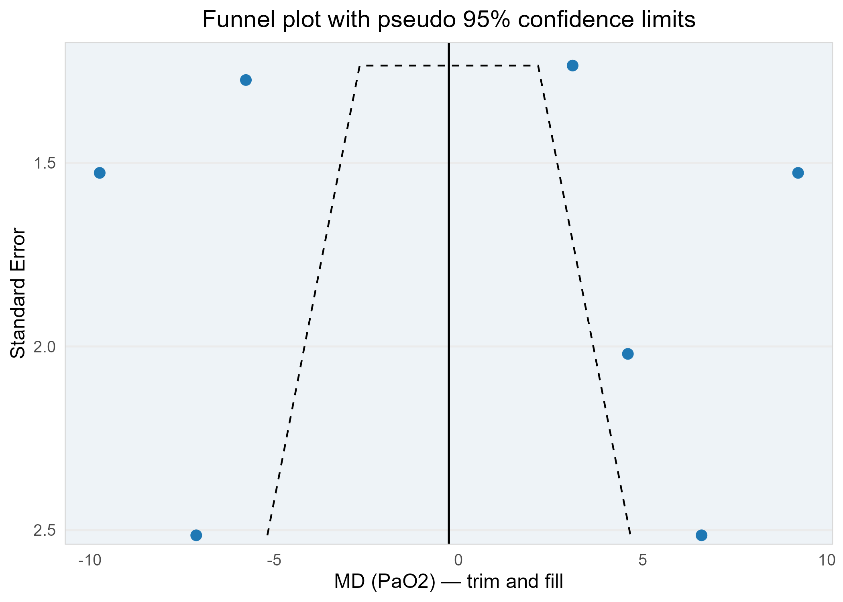


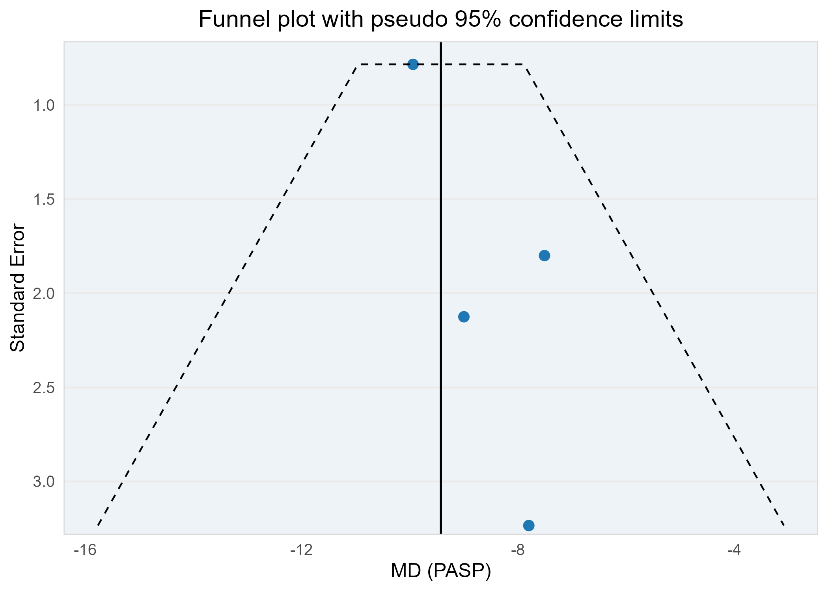


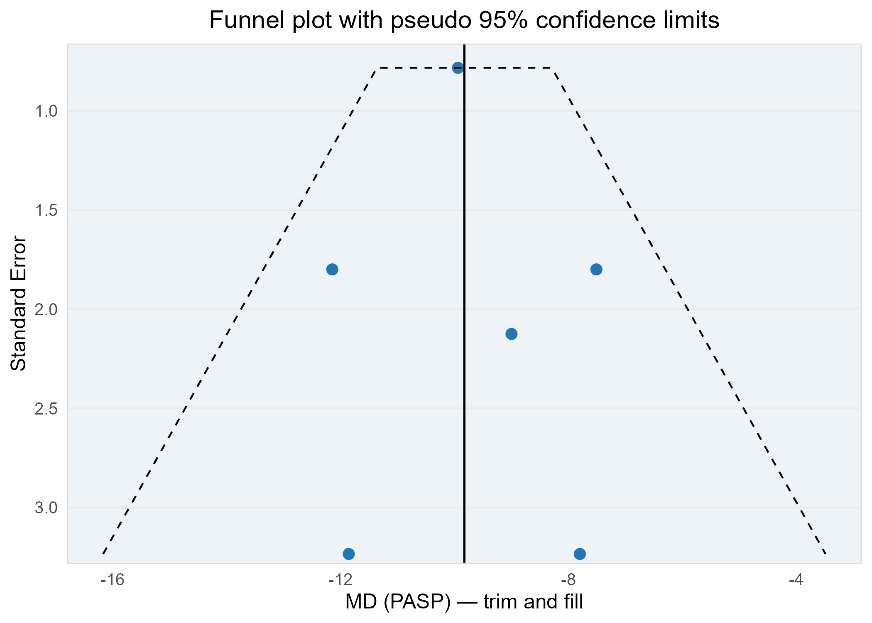


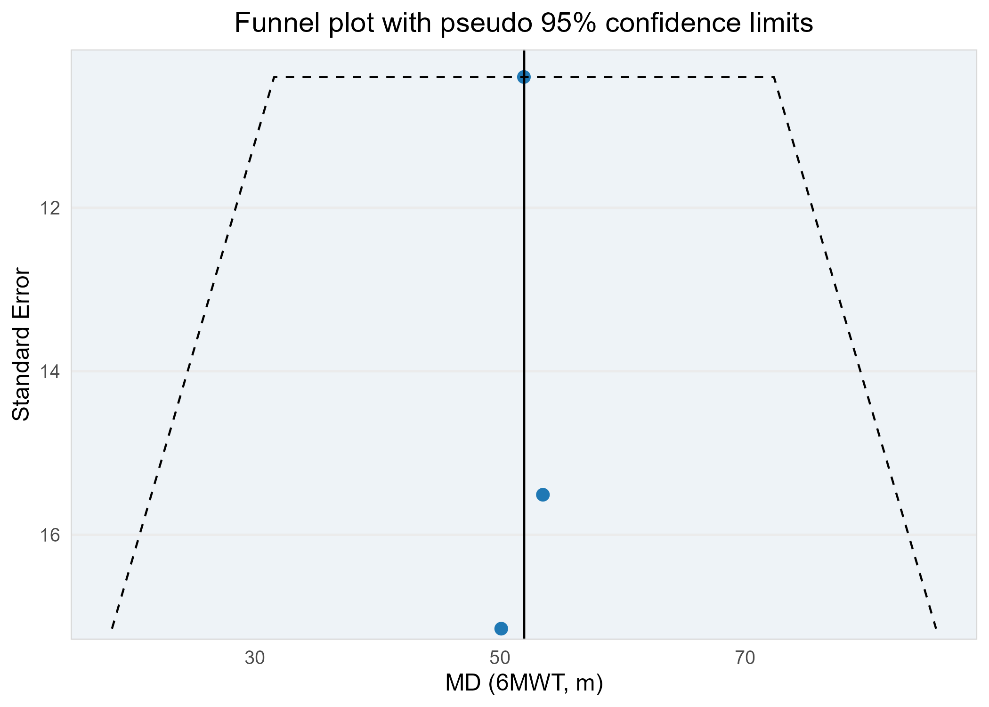


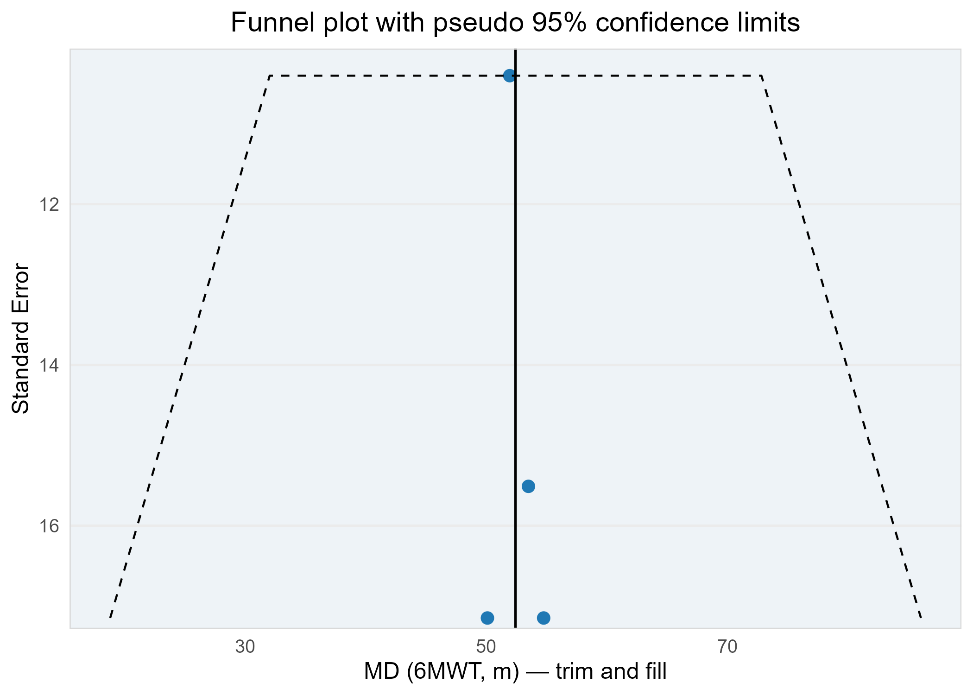


**Supplementary Figures 6-10. Sensitivity analyses and trim-and-fill adjustments.** (6-9) Sequential exclusion sensitivity analyses for each outcome demonstrating the influence of individual studies on pooled estimates. Horizontal lines represent 95% CI for pooled estimates after excluding each study. (10) Trim-and-fill analysis for PaO₂ showing one imputed study (hollow circle) on the left side, with adjusted pooled estimate (MD = 1.68 mmHg, 95% CI: 0.45 to 2.91) maintaining statistical significance despite correction for potential publication bias.
